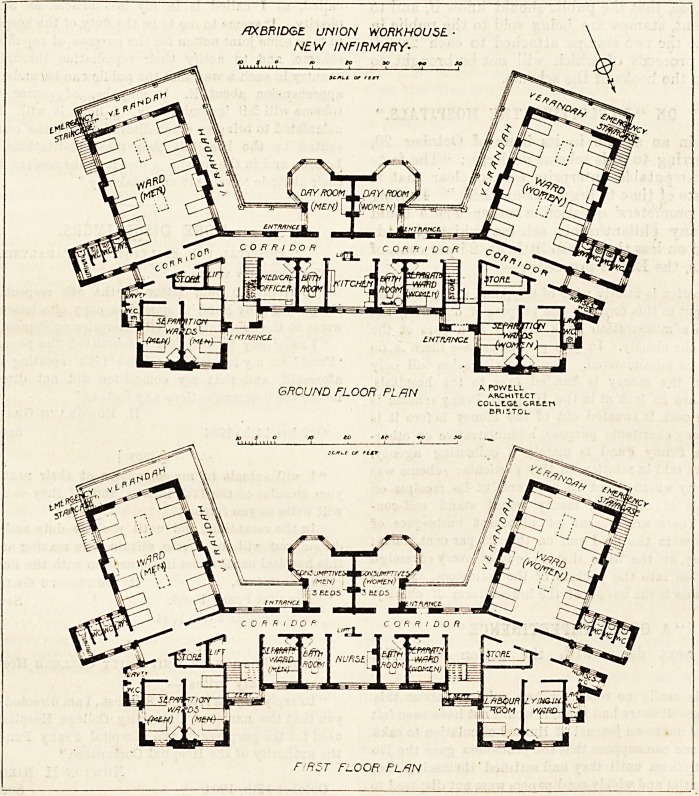# Axbridge Workhouse Infirmary

**Published:** 1904-10-22

**Authors:** 


### AXBRIDGE WORKHOUSE INFIRMARY.

For some years the Local Government Board have been pressing the Board of Guardians of the Axbridge Union to provide better accommodation for their sick and infirm patients, and after more than one scheme had been discussed and abandoned it was finally decided to buy a field lying to the north-east of the workhouse and thereon to erect a detached infirmary. Undoubtedly the guardians decided wisely in this matter. Plans were prepared and received the sanction of the Local Government Board two years ago. The building was finished during the present year.

In design the ground plan of the infirmary somewhat resembles the letter E—the left-hand side of the letter being to the north, and the right-hand side to the south; the top and lower parts of the letter being angled respectively towards the east and west. The ends of the main wards face the south-east and south-west, so that the aspect is quite good. These wards are 36 feet long and 24 feet wide, and, as they are intended for 12 beds each, the superficial space per bed is practically 100 feet. Assuming a ceiling height of 12 feet we get nearly 1,200 feet per bed, which for a workhouse infirmary must be pronounced to be a very fair allowance, as it rarely happens that special diseases are under treatment in these wards. The infirmary being two stories high, there are four of these large wards, and a verandah seven feet wide surrounds two of their aspects. There are escape staircases at the corners of these blocks. Projecting from the centre of the verandah are two day-rooms, having hexagonal bays. The sanitary blocks are attached to the north-east and north-west corners of the wards. The lavatory and closet accommodation are sufficient, but we cannot express approval of the manner in which these adjuncts are attached to the wards. They ought to have been separated by a short cross-ventilated passage. There should be no exception to the latter plan in an infirmary, and the method adopted at Axbridge necessitates the placing of a bed in the ward where it has no window on either side. That is a real evil.

A corridor runs along the backs of the wards and day-rooms, and in the centre of the north side of this corridor the kitchen is placed conveniently to the component parts of the building. We do not see any kitchen scullery. On one side of the kitchen is a bath-room for the men, and on the other side is one for the women. Next that for men is the medical officer's room, and next that for women is a single-bedded ward. Adjoining these are the staircases, stores, and lifts.

The entrances are north of the staircases, and projecting to the north are the separation wards. These are twin-wards, and on one side they are partly blocked by the main entrance, and on the other by the closets; and the latter, like those of the large wards, are not properly cut off from the main building. Each of these separation wards has a window in its end and another in the side; but we doubt whether these will be found sufficient, and proper cross-ventilation cannot be carried out unless there be openings in the partitions dividing the wards; even then it would only be partial. As the wards are intended for offensive cases we think this point ought to have been more carefully considered.

The first floor is a replica of the ground floor, but the day-rooms are here intended for three consumptive patients each, and the space over the kitchen is used as a nurses' sitting-room.

In general conception it may be said that the design is a good one for its purpose, and we much regret that the defects we have pointed out were not remedied when the infirmary was in plan only.

The wards are warmed by open fireplaces and by hot-water coils, and the electric light is used. The floors are laid with "Euboeolith." This is a new patent by Emil Sequin, and, so far, it is said to give every satisfaction.

The infirmary contains 72 beds; and the cost, including land, lighting, and contingent expenses, was a little under £100 a bed, which is a very moderate sum for the accommodation provided.

The architect was Mr. Arthur Powell of Bristol, the contractor was Mr. C. Addicott of Weston-super-Mare; Messrs. Barford and Perkins fitted up the heating apparatus, and Messrs. Witting, Eborall and Co., the electric plant.

The building was formally opened a few months since by the Right Hon. Walter Long. At the luncheon several speeches were made; and in one of them appears the extraordinary statement that "the ancients had no hospitals." If the speaker had visited Greece upwards of two thousand years ago he would have found many hospitals, and some of them with splendid wards and better adapted for certain diseases than any possessed by England at the present day.

## Figures and Tables

**Figure f1:**